# Identification of the functional role of peroxiredoxin 6 in the progression of breast cancer

**DOI:** 10.1186/bcr1789

**Published:** 2007-11-02

**Authors:** Xin-Zhong Chang, Da-Qiang Li, Yi-Feng Hou, Jiong Wu, Jin-Song Lu, Gen-Hong Di, Wei Jin, Zhou-Luo Ou, Zhen-Zhou Shen, Zhi-Ming Shao

**Affiliations:** 1 Breast Cancer Institute, Cancer Hospital, Department of Oncology, Shanghai Medical College, Institutes of Biomedical Science, Fudan University, Shanghai, 200032, People's Republic of China; 2 Tianjin Medical University Cancer Institute and Hospital, Hexi district, Tianjin, 300060, China

## Abstract

**Introduction:**

The molecular mechanisms involved in breast cancer metastasis still remain unclear to date. In our previous study, differential expression of peroxiredoxin 6 was found between the highly metastatic MDA-MB-435HM cells and their parental counterparts, MDA-MB-435 cells. In this study, we investigated the effects of peroxiredoxin 6 on the proliferation and metastatic potential of human breast cancer cells and their potential mechanism.

**Methods:**

Expression of peroxiredoxin 6 in the highly metastatic MDA-MB-231HM cells was investigated by RT-PCR, real-time PCR and western blot. A recombinant expression plasmid of the human peroxiredoxin 6 gene was constructed and transfected into MDA-MB-231 and MDA-MB-435 cells. The effects of peroxiredoxin 6 on the proliferation and invasion of MDA-MB-231 and MDA-MB-435 cells were investigated by the Cell Counting Kit-8 method, colony-formation assay, adhesion assay, flow cytometry and invasion assay *in vitro*. miRNA was used to downregulate the expression of peroxiredoxin 6. Genes related to the invasion and metastasis of cancer were determined by RT-PCR, real-time PCR and western blot. The tumorigenicity and spontaneously metastatic capability regulated by peroxiredoxin 6 were determined using an orthotopic xenograft tumor model in athymic mice.

**Results:**

Overexpression of peroxiredoxin 6 in MDA-MB-231HM cells compared with their parental counterparts was confirmed. Upregulation of peroxiredoxin 6 enhanced the *in vitro *proliferation and invasion of breast cancer cells. The enhancement was associated with decreasing levels of tissue inhibitor of matrix metalloproteinase (TIMP)-2 and increasing levels of the urokinase-type plasminogen activator receptor (uPAR), Ets-1 (E26 transformation-specific-1), matrix metalloproteinase (MMP)-9 and RhoC (ras homolog gene family, member C) expression. The results were further demonstrated by RNA interference experiments *in vitro*. In an *in vivo *study, we also demonstrated that peroxiredoxin 6-transfected breast cancer cells grew much faster and had more pulmonary metastases than control cells. By contrast, peroxiredoxin 6 knockdown breast cancer cells grew more slowly and had fewer pulmonary metastases. Effects similar to those of peroxiredoxin 6 on the uPAR, Ets-1, MMP-9, RhoC and TIMP-2 expression observed in *in vitro *studies were found in the *in vivo *study.

**Conclusion:**

Overexpression of peroxiredoxin 6 leads to a more invasive phenotype and metastatic potential in human breast cancer, at least in part, through regulation of the levels of uPAR, Ets-1, MMP-9, RhoC and TIMP-2 expression.

## Introduction

Breast cancer is the leading malignancy and also the leading cause of death from cancer in women [1]. The vast majority of these deaths are owing to metastasis. Although a number of significant advances have been made, the molecular mechanisms contributing to progression of breast cancer are poorly understood.

In our previous study, a novel breast cancer cell line that was derived from six cycles of pulmonary metastasis implantation into the mammary fat pad (MFP), designated MDA-MB-435HM (high metastasis (HM)), displayed significantly enhanced pulmonary metastatic potential compared with its parental counterparts, MDA-MB-435 cells [2]. Recently, another novel, highly metastatic MDA-MB-231HM cell line, derived from MDA-MB-231 cells, was also established and maintained using the same methods. These cells provide appropriate model systems, with a similar genetic background, for the comparative study of the molecular events involved in pulmonary metastasis of breast cancer.

In the postgenome era, global strategies are being developed to identify new components of complex biological processes, such as tumor cell metastasis. Proteomics enable the analysis of thousands of modified or unmodified proteins simultaneously and has become increasingly popular for identifying biomarkers for early detection, classification and prognosis of tumors, in addition to pinpointing targets for improved treatment outcomes. Our institute had used this approach to identify eleven proteins differentially expressed between the MDA-MB-435HM cells and their parental counterparts, MDA-MB-435 cells [2]. Some of these proteins, including peroxiredoxin (PRDX) 6, were not described previously in breast cancer metastasis. A significant association between PRDX6 staining and the presence of lymph-node metastasis in 80 breast cancer donors was also observed. However, we do not know whether PRDX6 is an instigator of metastasis or merely a correlative product during progression of breast cancer.

PRDX6 is a bifunctional 25 kDa protein with both GSH (Glutathione) peroxidase and phospholipase A_2 _activities. PRDX6 is expressed in many organs, with a particularly high level of expression in the lungs; its peroxidase activity was first demonstrated for protein isolated from the ciliary body of the bovine eye [3]. Subsequently, a human cDNA that encodes this protein was identified by random cloning [4]. The same protein was subsequently isolated from rat lung as a calcium (Ca^2+^)-independent phospholipase A_2_. Studies with recombinant proteins confirmed both Ca^2+^-independent phospholipase A_2 _[5] and peroxidase activities [6].

PRDX6 is an important antioxidant enzyme and has a major role in lung phospholipid metabolism. Manevich and Fisher found that PRDX6 was stably overexpressed in cells protected against oxidative stress, whereas antisense treatment resulted in oxidant stress and apoptosis [7]. They postulated that PRDX6 functions in antioxidant defense mainly by facilitated repair of damaged cell membranes through reduction of peroxidized phospholipids. The phospholipase A_2 _activity of PRDX6 is Ca^2+^-independent and maximal at acidic pH. Inhibition of phospholipase A_2 _activity results in alterations of lung-surfactant phospholipid synthesis and turnover. However, the role of PRDX6 in breast cancers remains unknown.

## Materials and methods

### Cell lines and animals

Human MDA-MB-435 and MDA-MB-231 breast cancer cell lines were bought from ATCC (American Type Culture Collection, Manassas, VA, USA). The highly metastatic MDA-MB-231HM cell line was established by our institute. Both MDA-MB-435 and MDA-MB-231 cells were routinely maintained in Leibovitz's L-15 medium with 2 mM of L-glutamine at 37°C in a humidified atmosphere containing 5% carbon dioxide (CO_2_). The medium was supplemented with 10% fetal bovine serum (FBS), 100 U/ml of penicillin and 100 μg/ml of streptomycin. The medium was changed every 2 to 3 days, and cells were subcultured by treatment with 0.25% trypsin/0.53 mM EDTA (ethylenediaminetetraacetic acid) solution. Female athymic BALB/c-*nu*/*nu *mice, 4 to 6 weeks' old, were obtained from the Shanghai Institute of Materia Medica of the Chinese Academy of Sciences (Shanghai, China) and housed in laminar-flow cabinets under specific pathogen-free conditions, with food and water *ad libitum*. All experiments on mice were conducted in accordance with the guidelines of National Institutes of Health (NIH; Bethesda, MD, USA) for the care and use of laboratory animals. The study protocol was also approved by Shanghai Medical Experimental Animal Care Committee (China).

### RT-PCR and western blot analysis

Total RNA was extracted from cultured cells using TRIzol^® ^Reagent (Invitrogen, San Diego, CA, USA) and RT-PCR was performed according to the manufacturer's instructions (MBI Fermentas, Vilnius, Lithuania). Gene-specific primers for the human PRDX6 gene (forward, 5'-TCATGGGGCATTCTCTTCTC-3'; reverse, 5'-TCTTCTTCAGGGATGGTTGG-3'; 497 bps) were synthesized at Shanghai Sangon Biological Engineering & Technology Services Co. (Shanghai, China). PCR products were separated on a 1.2% agarose gel and imaged on an Alpha Image 950 documentation system (Alpha Innotech, San Leandro, CA, USA). All experiments were repeated at least three times, and the GAPDH (glyceraldehyde-3-phosphate dehydrogenase) gene (forward, 5'-GGGAGCCAAAAGGGTCATCATCTC-3'; reverse, 5'-CCATGCCAGTGAGCTTCCCGTTC-3'; 353 bps) was chosen as an internal control.

To quantitatively determine the level of mRNA expression of the PRDX6, cDNA templates of the cells were amplified by real-time quantitative PCR. The reaction was performed using the DNA Engine Opticon™ 2 real-time PCR detection system (MJ Research, Watertown, MA, USA) with SYBR Green I (Molecular Probes Inc, Eugene, OR, USA). GAPDH was chosen as an internal control. The Opticon™ 2 apparatus was used to measure the fluorescence of each sample in every cycle at the end of the extension, and the comparative threshold cycle (2^-ΔΔ*cT*^) method was used to enable quantification of the mRNA of these genes. All samples were run in triplicate. After PCR, a melting curve was obtained and analyzed.

Western blot analysis was performed according to the previous method, with some modifications [8]. Briefly, proteins were extracted from the cultured cells and then quantitated using the bicinchoninic acid (BCA) assay kit (Pierce, Rockford, IL, USA), with BSA as a standard. Equal amounts of protein (50 μg) from different cells were separated by 10% SDS-PAGE and then incubated with mouse antihuman monoclonal antibodies against PRDX6 (Chemicon International Inc., Temecula, CA, USA). Target proteins were detected by the enhanced chemiluminescence (ECL) kit (Amersham Pharmacia Biotech, Uppsala, Sweden) and exposure to Biomax ML film (Eastman Kodak, Rochester, NY, USA). Images were captured by Alpha Image 950 documentation system and analyzed by NIH Image version 1.62 (Alpha Innotech, San Leandro, CA). The relative protein level in different cell lines was normalized to the signal intensity of β-actin, as an internal control.

### Construction of a human peroxiredoxin 6 expression vector and stable transfections

A human PRDX6 expression vector was constructed using the pcDNA™ 3.1 Directional TOPO^® ^Expression Kit (Invitrogen), according to the manufacturer's instructions. To construct the human PRDX6 expression vector, the entire open-reading frame (ORF) of the human PRDX6 gene (GenBank accession no. D14662) was amplified from the recombinant plasmid vector pBlueScript II SK(+), in which the full-length KIAA0106 cDNA had been inserted (kindly provided by Professor Takahiro Nagase, Kazusa DNA Research Institute, Chiba, Japan) by PCR using high-fidelity Platinum^® ^*Taq *DNA Polymerase (Invitrogen) and specific primers (upstream, 5'-CACCATGCCCGGAGGTCTGCTTCTC-3', with an added *CACC *underlined, according to the manufacturer's instructions; downstream, 5'-TTAAGGCTGGGGTGTGTAGCGGA-3'). The resulting construct was named 'pcDNA3.1D/PRDX6'. After identification by automated sequencing analysis, the recombinant DNA plasmids transfected breast cancer cells using Lipofectamine™ 2000 Transfection Reagent (Invitrogen), according to the manufacturer's instructions. The positive colonies were selected in the presence of 800 μg/ml of Geneticin^® ^(Invitrogen) and identified by RT-PCR and western blot analysis. In this study, the clones in which the PRDX6 gene were successfully transfected were named 'MDA-MB-435/PRDX6' and 'MDA-MB-231/PRDX6', respectively. The cells only transfected with the pcDNA3.1 vector were named 'MDA-MB-435/vector' and 'MDA-MB-231/vector'. The transfectants were used before passage 20 in all cases, to minimize the impacts of clonal diversification and phenotypic instability. For all functional and biological assays, cells between 70% and 90% confluence were used, with viability > 95%.

### Cell-proliferation assay

Cell-proliferation assays were performed to analyze the proliferation potential of parental, empty vector and PRDX6-transfected cells by using Cell-Counting Kit (CCK)-8 (Dojindo, Kumamoto, Japan). The cells were harvested and plated in 96-well plates at 1 × 10^3 ^cells/well and maintained at 37°C in a humidified incubator. At the indicated time points, 10 μl of the CCK-8 solution was added into the triplicate wells and incubated for 1.5 hours and the absorbance at 450 nm was measured to calculate the numbers of vital cells in each well.

### Colony-formation assay

The colony-formation assay was performed to measure growth promotion by PRDX6 transfection, according to the previously reported protocol [9], with some modifications. Identical numbers of parental cells, empty vector and PRDX6-transfected cells were seeded in six-well tissue-culture plates to form colonies. After 10 days' incubation, the number of colonies (≥20 cells) was counted within a field at a magnification of × 200 under a light microscope. For each test, a total of five fields were selected at random and the numbers were averaged. The assay was repeated in at least three independent experiments, with 0.5 × 10^4^, 1 × 10^4 ^and 2 × 10^4 ^of the cells seeded, respectively.

### Flow cytometry

The parental, empty vector and PRDX6-transfected cells at logarithmic growth phase were harvested and single-cell suspensions containing 1 × 10^6 ^cells were made. The target cells were treated following the standardized protocol and cell-cycle analyses were performed by flow cytometry.

### Adhesion assay

Matrigel™ (2 μg; BD Biosciences, San Jose, CA, USA) in 10 μl of serum-free culture medium was added to coat each well of a 96-well culture plate and then 20 μl of medium containing 3% BSA was added to each well. The wells were incubated at 37°C for 1 hour and washed twice with warm PBS. A 100 μl cell suspension that contained 5 × 10^4 ^cells was added to each well. After incubation for 1 hour, the wells were washed twice with PBS. We used the wells that were seeded with corresponding cells 6 hours previously as the control. The CCK-8 was used to detect the optical density (OD) value of each well. The adhesive rate was calculated according to the following formula: rate = (the trial OD ÷ the control OD) × 100%.

### *In vitro *invasion assays

*In vitro *invasion assays were performed to analyze the invasive potential of parental, vector and PRDX6-transfected cells using a Matrigel™ invasion chamber (Becton Dickinson Labware, Bedford, MA, USA), as described previously, with some modifications [10]. Each well insert was coated with 100 μl of a 1:3 dilution of Matrigel™ in serum-free culture medium. Then, a mixture of 200 μl of medium with 10% FBS, 200 μl of the supernatant of the corresponding cell culture and 200 μl of the supernatant of the NIH/3T3 cell culture was added to the lower chambers as a chemoattractant, and 1 × 10^5 ^cells in 250 μl of serum-free medium were added to the top of this Matrigel™ layer. The cells were incubated at 37°C for 24 hours. The cell suspension was aspirated and excess Matrigel™ was removed from the filter using a cotton swab. Then, the filters were fixed in 10% formalin solution and stained with H & E. Cells that had invaded through the Matrigel™ and reached the lower surface of the filter were counted under a light microscope at a magnification of × 200. Five fields should be counted for each sample.

### RNA interference experiments

To further demonstrate the role for PRDX6 in the progression of human breast cancer, we used the RNA interference (RNAi) technique to downregulate PRDX6 expression using the BLOCK-iT™ Pol II miR RNAi Expression Vector Kit (Invitrogen), according to the manufacturer's instructions.

The pcDNA™6.2-GW/EmGFP-miR plasmid with a spectinomycin-resistance gene (Invitrogen, San Diego, CA, USA) was used for cloning small synthetic oligonucleotides. Four different miR155-based PRDX6-targeting sequences were designed using Invitrogen's RNAi design algorithm online. These sequences are shown in Table 1. On the basis of the computer analysis, these inserted oligonucleotides would specifically bind homologous sites of PRDX6 mRNA and thus might interfere with PRDX6 expression in cultured cells. To identify successful construction of recombinant plasmids, the EmGFP (enhanced Emerald Green Fluorescent Protein) forward-sequencing primer (5'-GGCATGGACGAGCTGTACAA-3') and miRNA reverse-sequencing primer (5'-CTCTAGATCAACCACTTTGT-3') were designed to perform PCR. After PCR, the plasmids that contained the miRNA insert fragments were verified by DNA sequencing. Finally, the mutants were excluded from this experiment and the right one was purified using QIAGEN Plasmid Maxi (tip 500) Kit (QIAGEN, Hilden, Germany).

**Table 1:** The oligonucleotide sequences of shRNA (short hairpin RNA) driven by CMV(Cytomegalovirus) promoter in pcDNA™6.2-GW/EmGFP-miR (peroxiredoxin 6; GenBank accession no. D14662) (Invitrogen, San Diego, CA, USA)

	Top oligo	Bottom oligo
pCMV-PRDX6miRNA-122 (122–142 nt)	TGCTG*CCATGAGTCTCCCAGAAAGTC*GTTTTGGCCACTGACTGACGACTTTCTGAGACTCATGG	CCTGCCATGAGTCTCAGAAAGTCGTCAGTCAGTGGCCAAAAC*GACTTTCTGGGAGACTCATGG*C
pCMV-PRDX6miRNA-251 (251–271 nt)	TGCTG*AACACTGTCTATTGAAAGGGC*GTTTTGGCCACTGACTGACGCCCTTTCTAGACAGTGTT	CCTGAACACTGTCTAGAAAGGGCGTCAGTCAGTGGCCAAAAC*GCCCTTTCAATAGACAGTGTT*C
pCMV-PRDX6miRNA-289 (289–309 nt)	TGCTG*TAAGCATTGATATCCTTGCTC*GTTTTGGCCACTGACTGACGAGCAAGGATCAATGCTTA	CCTGTAAGCATTGATCCTTGCTCGTCAGTCAGTGGCCAAAAC*GAGCAAGGATATCAATGCTTA*C
pCMV-PRDX6miRNA-672 (672–692 nt)	TGCTG*ATTTCTTGCCAGATGGGAGCT*GTTTTGGCCACTGACTGACAGCTCCCATGGCAAGAAAT	CCTGATTTCTTGCCATGGGAGCTGTCAGTCAGTGGCCAAAAC*AGCTCCCATCTGGCAAGAAAT*C

#### Transfection of miRNA plasmids

The pcDNA™6.2-GW/EmGFP-miR expression vectors (Invitrogen, San Diego, CA, USA) containing either the PRDX6 miRNA insert (pCMV-PRDX6 miRNA-122, pCMV-PRDX6 miRNA-251, pCMV-PRDX6 miRNA-289 or pCMV-PRDX6 miRNA-672) or the pcDNA™6.2-GW/EmGFP-miR-neg control plasmid (Invitrogen) were transfected into target cells with the Lipofectamine 2000 reagent, according to the manufacturer's instructions. After 48 hours of transfection, RT-PCR, real-time PCR and western blot analysis were performed to assess the selectivity of PRDX6 knockdown. Successfully transfected cells clones were obtained by long-term culture in a selection medium containing 4 μg/ml of blasticidin. After 3 months, RT-PCR, real-time PCR and western blot analysis were performed to confirm the knockdown of PRDX6.

#### *In vitro *invasion assay

The effects of reduced PRDX6 expression on the invasion capacity of breast cancer cells were determined by *in vitro *invasion assay, as described previously. All experiments were performed in triplicate.

#### RT-PCR and western blot analysis

The levels of mRNA and protein expression of matrix metalloproteinase (MMP)-1, MMP-2, MMP-7, MMP-9, transcriptional factor Ets-1 (E26 transformation-specific-1), tissue inhibitor of MMP (TIMP)-1, TIMP-2, urokinase-type plasminogen activator (uPA), the uPA receptor (uPAR), cathepsin D, maspin, cystatin C, vascular endothelial growth factor (VEGF), basic fibroblast growth factor (bFGF), RhoC (ras homolog gene family, member C), P21, IGF(insulin-like growth factor)-1, IGF-1R (IGF-1 receptor), IGF-2, cyclin A, cyclin D1, cyclin E, E-cadherin, ps2, C-jun, C-fos, trophinin, tropomyosin (TPM)4 and TGF(transforming growth factor)-α in the control cells, PRDX6 transfectants and PRDX6-knockdown cells were determined by RT-PCR, real-time PCR and western blot analysis, respectively. The primers were designed using Primer 3 software (Whitehead Institute for Biomedical Research, Cambridge, MA, USA) (Additional file 1). All experiments were performed in triplicate.

### Tumorigenicity and metastasis assays in athymic mice

The tumorigenicity and spontaneously metastatic capability of the different cells were determined using an orthotopic xenograft tumor model in the athymic mice, as described previously [11]. Animals were divided into four groups: the parental, empty vector, PRDX6-transfected and pCMV-PRDX6 miRNA-672 (PRDX6 knockdown) groups. Each group had eight mice. MDA-MB-435 or MDA-MB-231 cells (5 × 10^6 ^or 2 × 10^6 ^cells, respectively) were injected orthotopically into the exposed axillary MFP of anesthetized athymic mice. The cells in PRDX6 knockdown groups were inoculated 4 weeks after transfection. Animals were monitored every 2 days for tumor growth and general health. The rate of primary tumor growth of different cells was determined by plotting the means of two orthogonal diameters of the tumors, measured at 7-day intervals. Animals were killed and autopsied at 8 weeks for MDA-MB-435 cells or 6 weeks for MDA-MB-231 cells postinoculation. The lungs used to evaluate the numbers of metastasis were fixed in Bouin's solution for 24 hours and then stored in 100% ethanol. When the lungs restored their inherent color, the white metastasis deposits could be assessed by macroscopic observation. To confirm the presence of lung metastases, sections were cut at 50 μm intervals and H & E staining was performed. In this study, the number of metastasis nodules on the lung surface was counted. Two independent pathologists calculated the number of metastases. Tissue samples harvested for western blot analysis were snap-frozen in liquid nitrogen.

### Western blot analysis of PRDX6, uPAR, Ets-1, MMP-9, RhoC and TIMP-2 expression in xenograft tumor tissues 

Proteins were extracted from the above frozen tissues and quantitated with the BCA assay kit. Western blot analysis was performed according to the methods described above. The relative protein expression of PRDX6, the uPAR, Ets-1, MMP-9, RhoC and TIMP-2 in xenograft tumor tissues of different groups was normalized to the signal intensity of β-actin.

### Statistical analysis

Statistical analysis was performed using the software of Statistical Package for the Social Sciences (SPSS) Version 11.5 for Windows (SPSS Inc., Chicago, IL, USA) and *P *< 0.05 was considered as statistically significant.

## Results

### Expression of peroxiredoxin 6 in different MDA-MB-231 cells

**Figure 1:**
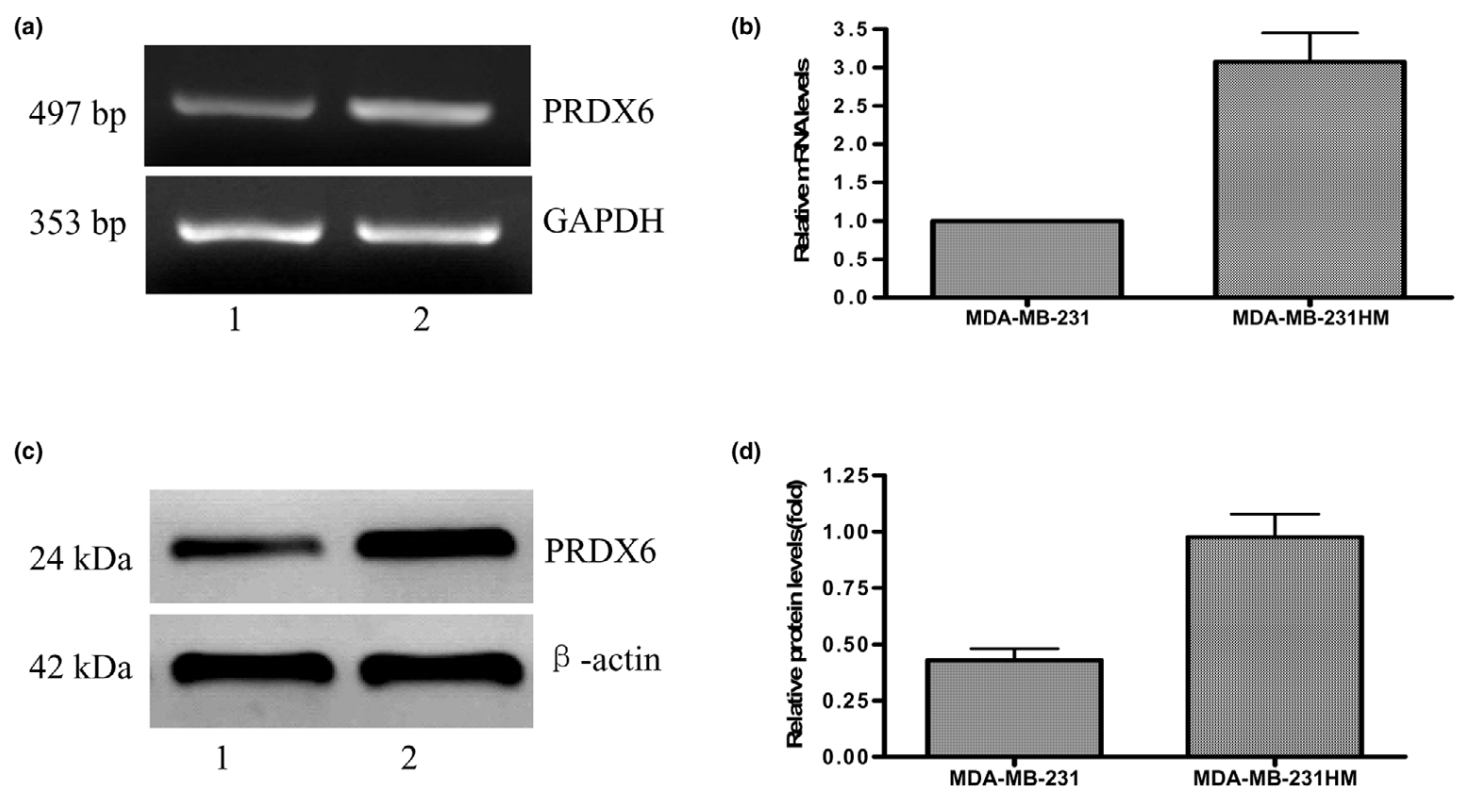
RT-PCR, real-time PCR and western blot analysis of PRDX6 differentially expressed in MDA-MB-231HM (lane 2) and parental MDA-MB-231 cells (lane 1). Differential expression of PRDX6 in both cell lines revealed by RT-PCR **(a) **and quantitative real-time PCR **(b) **analysis. **(c) **Representative immunodetection of PRDX6 is shown. **(d) **Relative protein expression of PRDX6 in different cell lines was normalized to the signal intensity of β-actin. PRDX, peroxiredoxin; GAPDH, glyceraldehyde-3-phosphate dehydrogenase.

To confirm the roles of PRDX6 in MDA-MB-231 cells, RT-PCR, real-time PCR and western blot analysis of PRDX6 were performed. As shown in Figure 1, results of replicate samples from MDA-MB-231HM and MDA-MB-231 cells confirmed a close correlation similar to that of MDA-MB-435 revealed by proteomic analysis [2], which indicated the functional relevance to breast cancer development and metastasis.

### *In vitro *effect of peroxiredoxin 6 overexpression on breast cancer cells

#### Stable transfection and expression analysis

**Figure 2:**
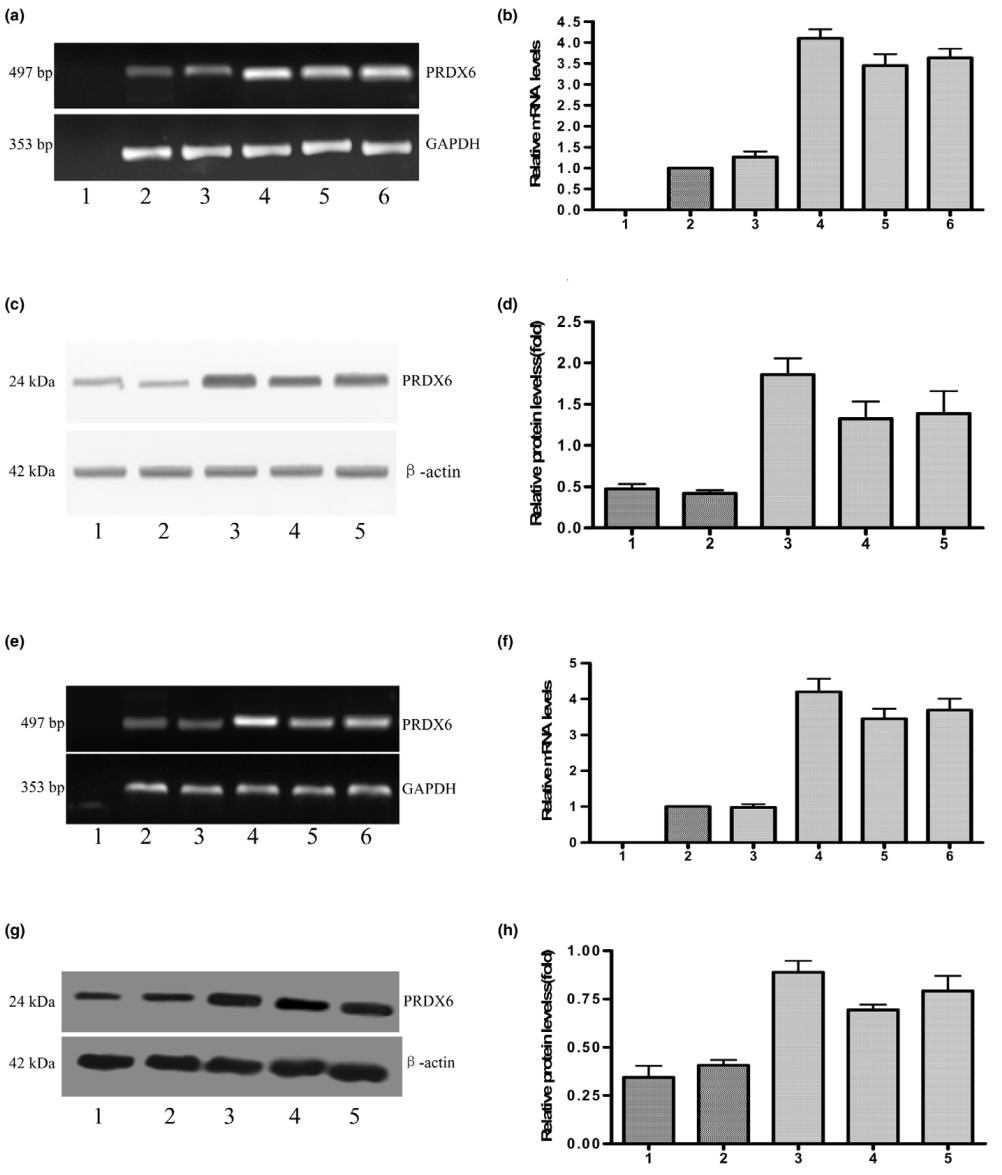
Expression of PRDX6 in PRDX6 transfectants and control cells. RT-PCR **(a) **and quantitative real-time PCR **(b) **analysis of PRDX6 expression in different MDA-MB-231 cells. Lanes 1 to 6: negative control (total RNA sample of MDA-MB-231HM cells without reverse transcription), MDA-MB-231, MDA-MB-231/vector, MDA-MB-231/PRDX6-1, MDA-MB-231/PRDX6-2 and MDA-MB-231/PRDX6-3.**(c) **Western blot analysis of PRDX6 expression in different MDA-MB-231 cells. Lanes 1 to 5: MDA-MB-231, MDA-MB-231/vector, MDA-MB-231/PRDX6-1, MDA-MB-231/PRDX6-2 and MDA-MB-231/PRDX6-3.**(d) **Relative protein expression of PRDX6 in different cell lines. RT-PCR **(e) **and quantitative real-time PCR **(f) **analysis of PRDX6 expression in different MDA-MB-435 cells. Lanes 1 to 6: negative control, MDA-MB-435, MDA-MB-435/vector, MDA-MB-435/PRDX6-1, MDA-MB-435/PRDX6-2 and MDA-MB-435/PRDX6-3. **(g) **Western blot analysis of PRDX6 expression in different MDA-MB-435 cells. Lanes 1 to 5: MDA-MB-435, MDA-MB-435/vector, MDA-MB-435/PRDX6-1, MDA-MB-435/PRDX6-2 and MDA-MB-435/PRDX6-3. **(h) **Relative protein expression of PRDX6 in different cell lines. PRDX, peroxiredoxin; GAPDH, glyceraldehyde-3-phosphate dehydrogenase.

A human PRDX6 expression vector was constructed and confirmed by automated DNA sequencing. To investigate the effects of PRDX6 on the metastatic phenotype of breast cancer cells, stable PRDX6 transfectants of MDA-MB-231 and MDA-MB-435 cells were established. As shown in Figure 2, RT-PCR, real-time PCR and western blot analysis revealed higher levels of PRDX6 expression in the two PRDX6 transfectants (MDA-MB-231/PRDX6-1 and MDA-MB-435/PRDX6-1) compared with the parental, vector control or other transfectant cells, respectively. In this study, both clones were selected as MDA-MB-231/PRDX6 and MDA-MB-435/PRDX6 for the subsequent experiments.

#### Cell-proliferation assay

**Figure 3:**
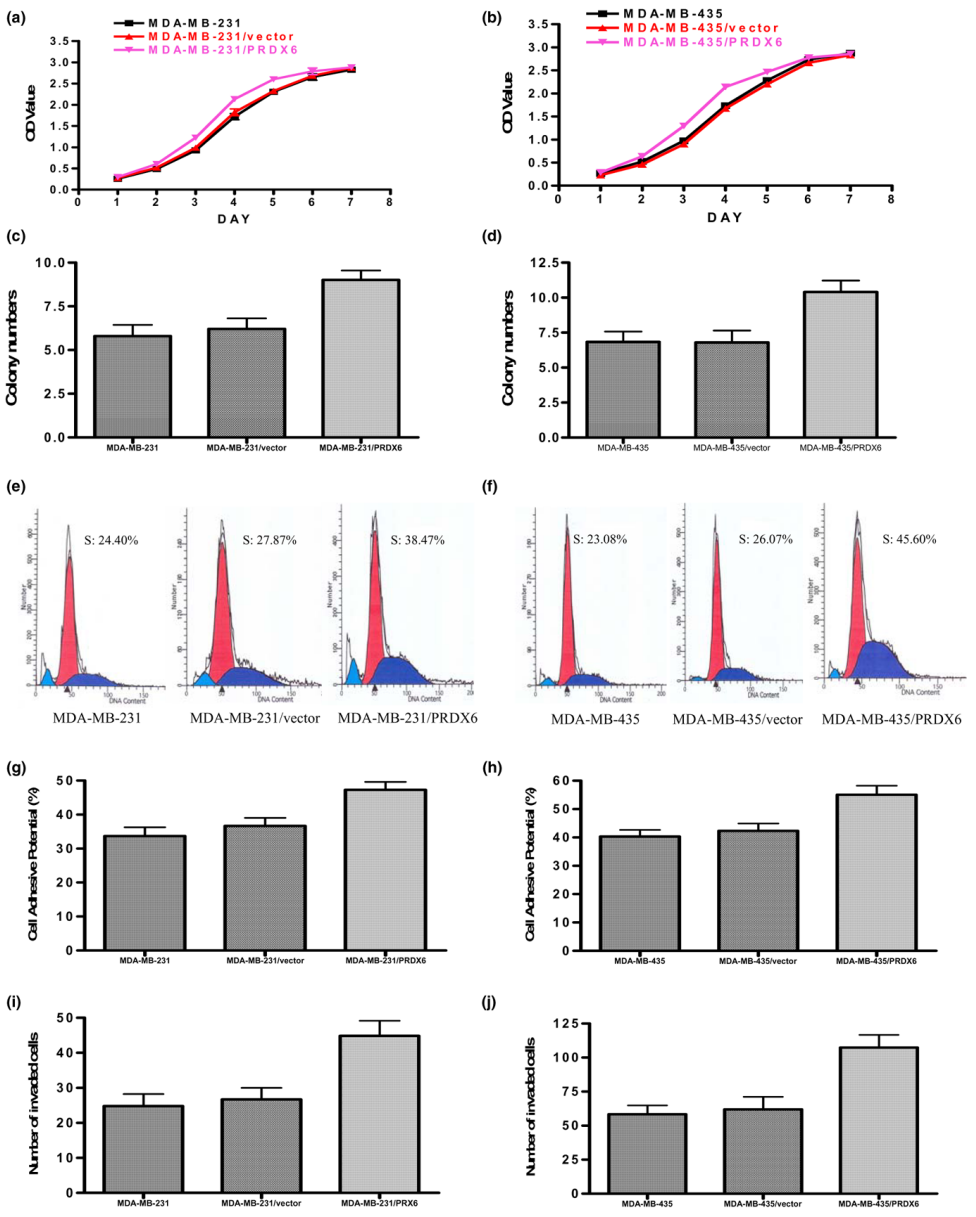
*In vitro *effects of PRDX6 overexpression on MDA-MB-231 cells. Growth curves for parent cells, empty vector and PRDX6-transfected cells in an *in vitro *proliferation assay for MDA-MB-231 **(a) **and MDA-MB-435 **(b) **cells. Colony-formation assay in the group of 1 × 10^4 ^cells for MDA-MB-231 **(c) **and MDA-MB-435 **(d) **cells. Representative results of flow-cytometric analysis for MDA-MB-231 **(e) **and MDA-MB-435 **(f) **cells. The percentage of PRDX6-transfected cells in the S-phase was higher than that in the two control cells. The cell-adhesive potential of different cells was compared for MDA-MB-231 **(g) **and MDA-MB-435 **(h) **cells. The potential of PRDX6-transfected cells for adhesion was higher than that of the two control cells. *In vitro *Matrigel™ invasion assay (BD Biosciences Discovery Labware, Bedford, MA, USA) for MDA-MB-231 **(i) **and MDA-MB-435 **(j) **cells. The PRDX6-transfected cells were more invasive than their parental counterparts (*P *< 0.05). OD, optical density; PRDX, peroxiredoxin.

We assessed the growth of the parental, PRDX6-transfected and vector-transfected cells. As shown in Figure 3a,b, PRDX6 was able to increase the proliferation of MDA-MB-231 and MDA-MB-435 cells significantly at day 3, 4 and 5 (*P *< 0.05).

#### Colony-formation assay

In all three groups, PRDX6-transfected cells formed more colonies than the control, but a statistically significant difference was only observed in the group of 1 × 10^4 ^cells (*P *< 0.05; Figure 3c,d).

#### Flow cytometry

Analysis of the cell cycle by flow cytometry also showed that the percentage of S-phase PRDX6-transfected cells was higher than that of the control cells (*P *< 0.05; Figure 3e,f).

#### Adhesion assay

The cell-adhesive ability was one of the key determinants of tumor metastasis. We examined the potential of different cells to adhere to Matrigel™ using the CCK-8 assay. As shown in Figure 3g,h, the values of transfected cells were higher than those of the control (*P *< 0.05).

#### *In vitro *invasion assays

*In vitro *invasion assays were performed to determine the effect of PRDX6 on cell invasion using the Boyden chamber assay (BD Biosciences Discovery Labware, Bedford, MA, USA). The Matrigel™ matrix served as a reconstituted basement membrane *in vitro*. The number of cells migrating through the Matrigel™ matrix was counted and the result is presented in Figure 3i,j. The PRDX6 transfectants showed higher invasive capacity than either parental or empty vector cells (*P *< 0.05).

### RNA interference experiments

#### miRNA constructs inhibited peroxiredoxin 6 expression

**Figure 4:**
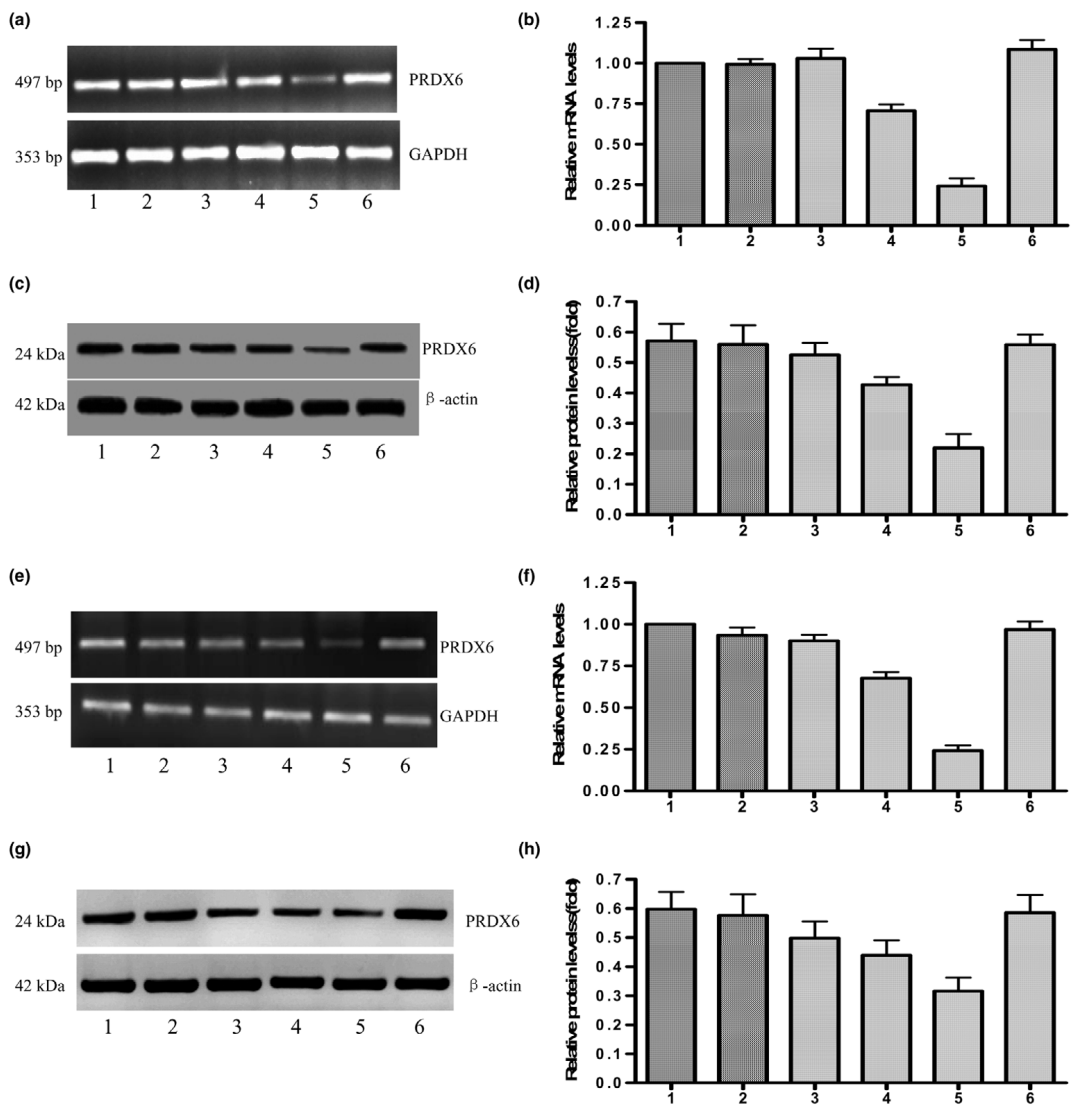
Transfection with pCMV-PRDX6 miRNA efficiently silences PRDX6 expression in breast cancer cells. Lanes 1 to 6: the parents, pCMV-PRDX6 miRNA-122-transfected, pCMV-PRDX6 miRNA-251-transfected, pCMV-PRDX6 miRNA-289-transfected, pCMV-PRDX6 miRNA-672-transfected and pCMV-PRDX6 miRNA-neg-transfected cells. RT-PCR **(a) **and real-time PCR **(b) **illustrated that pCMV-PRDX6 miRNA-672, rather than pCMV-PRDX6 miRNA-neg, pCMV-PRDX6 miRNA-122, pCMV-PRDX6 miRNA-251 or pCMV-PRDX6 miRNA-289, reduced PRDX6 mRNA in MDA-MB-231 cells. **(c) **A representative western blot image illustrated that pCMV-PRDX6 miRNA-672, rather than the other miRNA groups, reduced PRDX6 proteins in MDA-MB-231 cells.**(d) **The relative expression of PRDX6 protein in the different MDA-MB-231 cells above. RT-PCR **(e) **and real-time PCR **(f) **illustrated that pCMV-PRDX6 miRNA-672, rather than the other miRNA groups, reduced PRDX6 mRNA in MDA-MB-435 cells. **(g) **A representative western blot image illustrated that pCMV-PRDX6 miRNA-672, rather than the other miRNA groups, reduced the levels of PRDX6 protein in MDA-MB-435 cells. **(h) **The relative expressions of PRDX6 protein in the different MDA-MB-435 cells above. PRDX, peroxiredoxin; GAPDH, glyceraldehyde-3-phosphate dehydrogenase.

After PCR and DNA sequencing, the four recombinant plasmids targeting PRDX6 (pCMV-PRDX6 miRNA-122, pCMV-PRDX6 miRNA-251, pCMV-PRDX6 miRNA-289 and pCMV-PRDX6 miRNA-672) were transfected into the MDA-MB-231 and MDA-MB-435 cells, respectively. To evaluate inhibition of PRDX6, RT-PCR, real-time PCR and western blot analysis were performed to compare the expression levels of PRDX6 among the parental, pCMV-PRDX6 miRNA-122-transfected, pCMV-PRDX6 miRNA-251-transfected, pCMV-PRDX6 miRNA-289-transfected, pCMV-PRDX6 miRNA-672-transfected and pCMV-PRDX6 miRNA-neg-transfected cells 48 hours after transfection. As shown in Figure 4, PRDX6 was strongly inhibited in pCMV-PRDX6 miRNA-672-transfected cells.

To assess the phenotype of tumor cells in which PRDX6 expression was inhibited over a long-term period, we generated stable PRDX6-downregulated clonal cell lines. One of clones from pCMV-PRDX6 miRNA-672-transfected cells was chosen and cultured for a long-term period in a selection medium containing blasticidin. At 3 months, RT-PCR, realtime PCR and western blot analysis of the clone cells demonstrated decreased PRDX6 expression compared with parental cells (*P *< 0.05; Additional file 2).

#### Peroxiredoxin 6 knockdown inhibited breast cancer cell invasion *in vitro*

**Figure 5:**
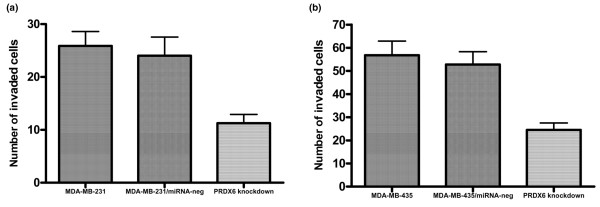
Downregulation of PRDX6 in breast cancer cells decreased the invasion capacity *in vitro*. *In vitro *invasion assays of the invasive potential of the different MDA-MB-231 **(a) **and MDA-MB-435 cells **(b)**. PRDX, peroxiredoxin.

pCMV-PRDX6 miRNA-672 was used to knockdown PRDX6 in MDA-MB-231 and MDA-MB-435 cells, to determine whether PRDX6 expression was crucial to the invasive phenotype *in vitro*. As shown in Figure 5, reduced PRDX6 expression in both cell types significantly inhibited the invasive capacity *in vitro *compared with parental and nonsilencing control cells (*P *< 0.05).

PRDX6 regulates uPAR, Ets-1, MMP-9, RhoC and TIMP-2 expression To further explore the molecular mechanisms underlying the PRDX6-mediated invasion phenotype *in vitro*, we focused on the several recognized invasion-associated genes. RT-PCR, real-time PCR and western blot analysis showed that PRDX6 transfectants constitutively express high levels of the uPAR, transcriptional factor Ets-1, MMP-9 and RhoC and low levels of TIMP-2 compared with control vector-transfected or parental cells. In the PRDX6 knockdown cells, the uPAR, Ets-1, MMP-9 and RhoC were downregulated and TIMP-2 was upregulated (Additional file 3). However, we did not find any significant differences in MMP-1, MMP-2, MMP-7, TIMP-1, uPA, cathepsin D, maspin, cystatin C, VEGF, bFGF, P21, IGF-1, IGF-1R, IGF-2, cyclin A, cyclin D1, cyclin E, E-cadherin, ps2, C-jun, C-fos, trophinin, TPM4 and TGF-α expression among the parental, PRDX6 transfectants, PRDX6 knockdown and control vector cells (data not shown).

### Tumor growth and pulmonary metastasis in the athymic mice were regulated by the expression of peroxiredoxin 6

**Figure 6:**
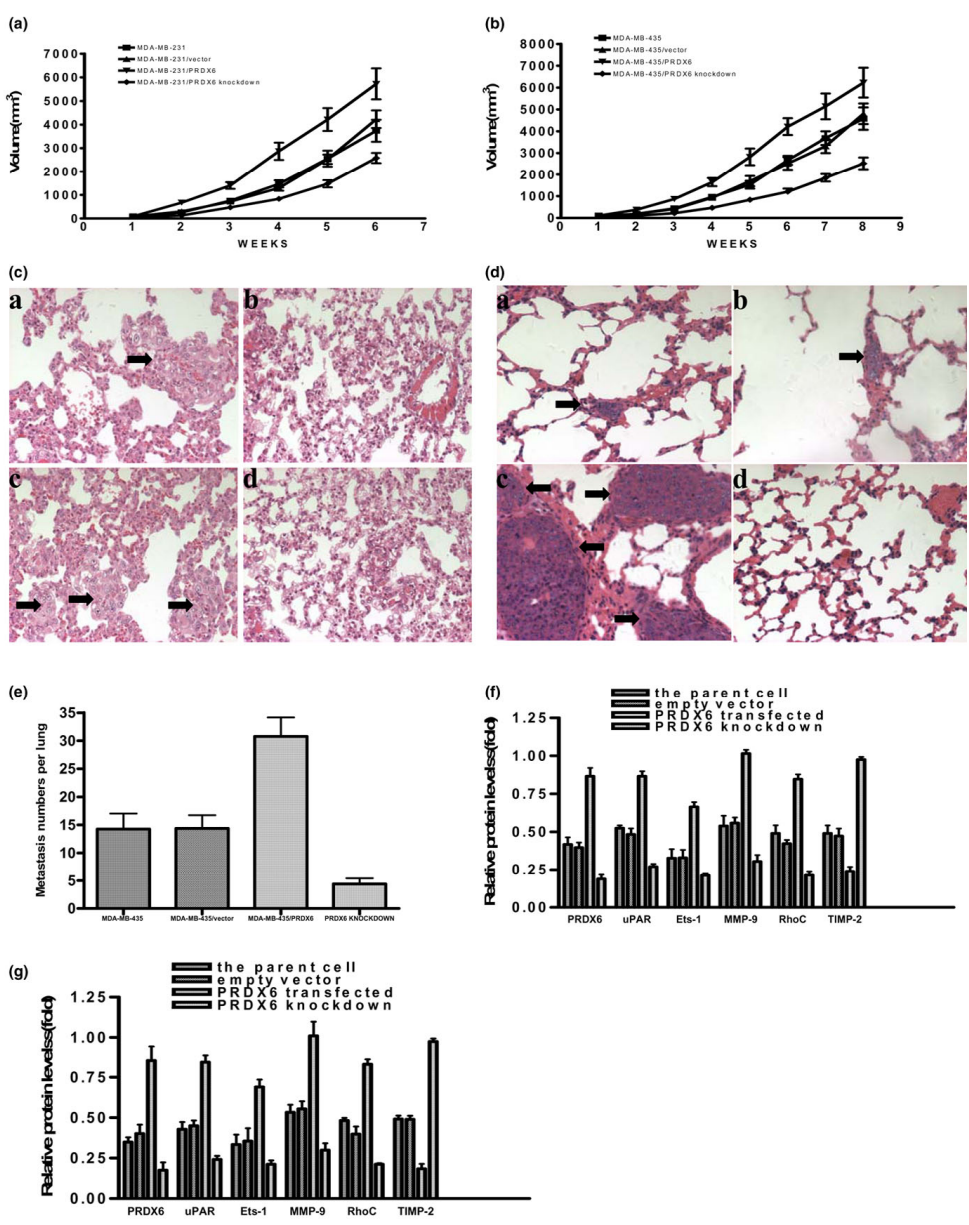
Regulation of the growth and pulmonary metastasis of breast cancer cells by PRDX6 in *in vivo *studies. The growth curves for different MDA-MB-231 **(a) **and MDA-MB-435 **(b) **cells in an *in vivo *proliferation assay. Photomicrographs of micrometastases (arrow) in lung sections obtained from mice bearing parental (*a*), vector-transfected (*b*), PRDX6-transfected (*c*) and PRDX6 knockdown (*d*) groups for MDA-MB-231 **(c) **and MDA-MB-435 **(d) **cells (H & E; × 200). **(e) **Metastasis numbers per lung in parental, vector-transfected, PRDX6-transfected and PRDX6 knockdown groups at week 8 for MDA-MB-435 cells. The relative protein expression of PRDX6, uPAR, Ets-1, MMP-9, RhoC and TIMP-2 was normalized to the signal intensity of β-actin for MDA-MB-231 **(f) **and MDA-MB-435 **(g) **cells. MMP, matrix metalloproteinase; PRDX, peroxiredoxin; TIMP, tissue inhibitor of MMP; uPAR, urokinase-type plasminogen activator receptor; Ets-1, E26 transformation-specific-1; RhoC, ras homolog gene family member C.

The effect of PRDX6 expression on tumor growth and metastasis was further assayed in athymic mice. Results revealed that PRDX6-transfected groups grew faster than either vector-transfected or parental groups and PRDX6 knockdown groups grew slower than the other three groups (Figure 6a,b). To study pulmonary metastasis, at the experimental end point, lungs were examined physically at autopsy and overt surface metastases were observed. For MDA-MB-231 cells, surface metastases were found in half of mice in the PRDX6-transfected groups, 12.5% of the parental group and none of the vector-transfected and PRDX6 knockdown groups. The difference between the PRDX6-transfected and the PRDX6 knockdown groups was significant (*P *< 0.05). Because MDA-MB-231 cells have a low potential for lung metastasis, we did not find a significant difference in lung metastases between the other groups. Furthermore, lungs were subjected to microscopic examination for morphologic evidence of tumor cells on H & E-stained paraffin sections. The most micrometastases were observed in PRDX6-transfected mice. Only a few or no micrometastases were observed in mice of parental, vector-transfected or knockdown groups (Figure 6c). For MDA-MB-435 cells, the most micrometastases were observed in the lungs of the PRDX6-transfected group, whereas the PRDX6 knockdown mice had the fewest micrometastases (Figure 6d). The number of overt surface metastases in the lungs of the PRDX6-transfected group was largest and that of the PRDX6 knockdown group was smallest (Figure 6e). The difference between the PRDX6-transfected or knockdown groups and the parental group was significant (*P *< 0.05). In addition, effects similar to those of PRDX6 on uPAR, Ets-1, MMP-9, RhoC and TIMP-2 expression observed in the *in vitro *studies were also found in the *in vivo *studies (Figure 6f–g).

## Discussion

Recently, many metastasis-related proteins have been found in different kinds of tumors by using the proteomic approach. In our institute, Li and colleagues have identified 11 proteins with significantly different expression levels in the high–metastatic-potential MDA-MB-435HM cells and their lower-metastatic-potential parental counterpart by using a comparative proteomic approach [2]. PRDX6 is one of the proteins with different levels of expression.

PRDXs represent a superfamily of Se(selenium)-independent peroxidases and some studies have showed a significant relationship between the expression of PRDXs and breast cancer. Noh and colleagues [12] reported that PRDX1, PRDX2 and PRDX3 were overexpressed in breast cancer tissues, suggesting that PRDXs had a proliferative effect and might be related to cancer development or progression. Karihtala and colleagues [13] observed that staining for PRDX2 and PRDX6 was negative in half of breast cancer cases, whereas PRDX1, PRDX3, PRDX4 and PRDX5 showed stronger expression levels. They also found that expression of PRDX5 was significantly stronger if tumors were larger or had lymph-node metastases. Liu and colleagues [14] reported that in a MDR (multidrug resistant) cell line MCF7/AdVp3000 the expression of PRDX6 was increased and the expression of PRDX2 was decreased, which suggested they might cause drug resistance to chemotherapy in breast cancer. In addition, the overexpression of PRDX2, resulting in increased resistance of breast cancer cells to ionizing radiation [15], and involvement of PRDX in breast cancer resistance to docetaxel therapy were also observed [16].

The PRDX6 gene located at chromosome 1q2 consists of five exons and encodes a 25 kDa protein. There have been numerous reports documenting a link between PRDX6 and certain diseases, such as atherosclerosis [17], sporadic Creutzfeldt–Jacob disease [18] and Pick disease [19]. However, until now, there was little information about the role of PRDX6 in the metastasis of cancers, including breast cancer.

In the present study, we found that PRDX6 was upregulated in MDA-MB-231HM cells. These results are consistent with the previous results in MDA-MB-435HM cells, suggesting that expression of PRDX6 takes part in the process of invasion and metastasis of breast cancer. We also established the PRDX6-overexpressing MDA-MB-231 and MDA-MB-435 cell lines by stable transfection. Both transfected cell types exhibited the significantly enhanced proliferative potential compared with their parental cells. The results of adhesion and invasive assays *in vitro *also showed that PRDX6 could promote the invasion and migration of breast cancer cells. This provided direct evidence that PRDX6 could enhance the invasive potential of breast cancer.

At first glance, our results seem to contradict some of the published literature regarding the relationship between PRDXs and cancers. Ouyang and colleagues [20] found that progression of prostate adenocarcinoma was correlated to the deregulation of antioxidants, including PRDX6, in Nkx3.1 mutant mice. They thought that the deregulation of antioxidants led to an accumulation of reactive oxygen species (ROS) and, ultimately, the initiation and progression of prostate carcinoma. This result was verified in some other studies. Neumann and colleagues [21] found that mice lacking PRDX1 have a shortened lifespan partly owing to several malignant cancers, including breast cancer. de Souza and colleagues observed decreases in the expression of PRDX2 and PRDX6 proteins [22] in murine melanoma line Tm1 compared with Tm5 from nontumor cells.

However, some other studies showed upregulation of PRDX6 in malignant tumors. Karihtala and colleagues [13] found that PRDXs, including PRDX6, were overexpressed in breast cancer. Quan and colleagues [23] demonstrated that enhanced PRDX6 expression was strongly associated with bladder cancer development. Furthermore, overexpression of PRDX6 was found in malignant mesothelioma of the lung [24].

Why is there such disparity in the literature regarding PRDX6 expression in various studies? One possibility might be differences in cell types. Most of the reports about the downregulation of PRDXs originated from studies in murine malignant cells. Another possible explanation for increased PRDX levels in tumors would be that they were the results of responding to impending oxidative stress and that antiapoptotic features of PRDXs provided a growth advantage to tumor cells [13]. It was probable that PRDXs were able to inhibit ROS-mediated physiological apoptosis, cause abnormal proliferation and thereby lead to tumorigenesis. However, for the deregulation of PRDXs in some malignant cells, a lack of PRDX led to an accumulation of ROS, which promoted carcinogenesis in all its stages, for example initiation, promotion and progression [25]. Clearly, further studies should be done to explain these contradictions.

In the present study, we also used miRNA-induced gene silencing to further demonstrate the relationship between invasive potential and expression of PRDX6. A miRNA expression vector derived from a murine miR-155 sequence, which enables the expression of engineered miRNA sequences from Pol II promoters, was used to construct a PRDX6-targeting miRNA expression vector. Four different vectors, pCMV-PRDX6 miRNA-122, pCMV-PRDX6 miRNA-251, pCMV-PRDX6 miRNA-289 and pCMV-PRDX6 miRNA-672, were constructed in this study. We found that the expression of PRDX6 was significantly decreased in pCMV-PRDX6 miRNA-672 groups of breast cancer cells. To confirm their stable nature for future inoculation *in vivo*, we generated stable transfectants of PRDX6 knockdown and the obvious inhibition was observed at 3 months. We found that PRDX6 knockdown cells exhibited marked inhibition in invasive potential and the inhibition might be related to the regulation of uPAR, Ets-1, MMP-9, RhoC and TIMP-2 levels.

A crucial step during invasion and metastasis is the destruction of biological barriers, such as the basement membrane, which requires activation of proteolytic enzymes. Many studies have shown that enhanced production of members of the plasminogen activator pathway and MMP family contributed to tumor invasion, metastasis and angiogenesis [26]. The uPAR could regulate cell-surface-associated proteolysis by uPA [27] and it was also involved in the regulation of cell adhesion and migration independent of the enzymatic activity of its ligand [28]. In this study, we have demonstrated that upregulated expression of the uPAR, but not uPA, was associated with increased tumor cell invasion and metastasis in breast cancer by PRDX6. By contrast, downregulated expression of the uPAR was associated with decreased tumor cell invasion and metastasis.

MMP activity is tightly regulated by specific physiological inhibitors, TIMPs [29]. The TIMP family comprises at least four distinct members: TIMP-1, TIMP-2, TIMP-3 and TIMP-4. Ets-1 is a member of the Ets family of transcription factors. It has been reported that Ets-1 was overexpressed in a variety of human malignancies, including breast cancers [30]. Because of its roles in the transcriptional regulation of MMPs, Ets-1 is a candidate mediator of cancer invasion and metastasis. In the present study, we demonstrated that the enhancement of invasive phenotype of breast cancer cells by PRDX6 was accompanied by upregulation of MMP-9 and Ets-1 expression and downregulation of TIMP-2 expression. However, no significant differences in expression of other members of the MMP and TIMP families were found among PRDX6-transfected, knockdown and parental cells. This suggested that the upregulation of MMP-9 might be stimulated by PRDX6 through activation of Ets-1 and deactivation of TIMP-2.

RhoC is a member of the Ras-homology family of small GTP(guanosine triphosphate)-binding proteins. Some studies have proved that RhoC had a key role in metastasis of breast cancer [31]. In this study, our results suggested that the effect of PRDX6 on the invasive and metastatic potential of breast cancer cells was mediated partially through regulation of RhoC expression.

In this study, we also demonstrated that PRDX6 enhanced orthotopic tumor growth and pulmonary metastasis in nude mice. By contrast, PRDX6 knockdown cells showed decreased ability in breast cancer growth, invasiveness, and metastasis. These changes were associated with regulation of MMP-9, Est-1, the uPAR, Rhoc and TIMP-2 proteins in xenograft tumors. All these results indicate that PRDX6 promotes breast cancer progression, partially through upregulation of uPAR, Ets-1, MMP-9 and RhoC expression and downregulation of TIMP-2 expression.

## Conclusion

We identified upregulation of PRDX6 in highly invasive and potentially metastatic MDA-MB-231HM breast cancer cells compared with their parental cells. Furthermore, we demonstrated that overexpression of PRDX6 in breast cancer cells promoted their invasive and metastatic potential *in vitro *and *in vivo*. Conversely, experimental inhibition of PRDX6 expression decreased the invasive and potential potential of breast cancer cells, partially through regulation of uPAR, Ets-1, MMP-9, RhoC and TIMP-2 expression. These results are consistent with our previous hypothesis that PRDX6 expression contributes to cellular invasive and metastatic potential.

## Abbreviations

BCA = bicinchoninic acid; bFGF = basic fibroblast growth factor; bp = base pair; BSA = bovone serum albumin; Ca^2+ ^= calcium; CCK = Cell-Counting Kit; CO_2 _= carbon dioxide; ECL = enhanced chemiluminescence; EDTA = ethylenediaminetetraacetic acid; Ets-1 = E26 transformation-specific-1; EmGFP = enhanced Emerald Green Fluorescent Protein; FBS = fetal bovine serum; GAPDH = glyceraldehyde-3-phosphate dehydrogenase; GSH = Glutathione; H&E = hematoxylin and eosin; HM = high metastasis; IGF = insulin-like growth factor; IGF-1R = IGF-1 receptor; MFP = mammary fat pad; miRNA, microRNA; MMP = matrix metalloproteinase; NIH = National Institutes of Health; OD = optical density; ORF = open-reading frame; PBS = phosphate-buffered saline; PCR = polymerase chain reaction; PRDX = peroxiredoxin; RhoC = ras homolog gene family, member C; ROS = reactive oxygen species; RNAi = RNA interference; RT-PCR = reverse-transcriptase PCR; Se = Selenium; TGF = transforming growth factor; TIMP = tissue inhibitor of MMP; TPM = tropomyosin; uPA = urokinase-type plasminogen activator; uPAR = uPA receptor; VEGF = vascular endothelial growth factor.

## Competing interests

The authors declare that they have no competing interests.

## Authors' contributions

XZC performed the experiments. ZMS was the principal investigator.

## Supplementary Material

Additional file 1 Table listing primers for RT-PCR and their annealing temperatures. Click here for file

Additional file 2 Figure showing that peroxiredoxin (PRDX) 6 was inhibited in breast cancer cells at 3 months after initial transfection. RT-PCR **(a) **and real-time PCR **(b) **showed that pCMV-PRDX6 miRNA-672 decreased PRDX6 mRNA in MDA-MB-231 cells at 3 months after initial transfection. (**C) **Western blot analysis of PRDX6 in different MDA-MB-231 cells at 3 months after initial transfection. **(d) **The relative protein expressions of PRDX6 showed that pCMV-PRDX6 miRNA-672 decreased the PRDX6 protein in MDA-MB-231 cells at 3 months after initial transfection. RT-PCR **(e) **and real-time PCR **(f) **illustrated that pCMV-PRDX6 miRNA-672 decreased PRDX6 mRNA in MDA-MB-435 cells at 3 months after initial transfection. **(g) **Western blot analysis of PRDX6 in different MDA-MB-435 cells. **(h) **The relative protein expressions of PRDX6 showed that pCMV-PRDX6 miRNA-672 decreased PRDX6 protein in MDA-MB-435 cells. Click here for file

Additional file 3 Expression of peroxiredoxin (PRDX) 6 in breast cancer cells regulated urokinase-type plasminogen activator (uPA) receptor (uPAR), Ets-1, matrix metalloproteinase (MMP)-9, RhoC and tissue inhibitor of MMP (TIMP)-2 expression in vitro. Lanes 1 to 5: parental, empty vector, PRDX6-transfected, pCMV-PRDX6 miRNA-neg and PRDX6 knockdown cells. RT-PCR (a) and quantitative real-time PCR (b) analysis of uPAR, Ets-1, MMP-9, RhoC and TIMP-2 mRNA expression regulated by PRDX6 in MDA-MB-231 cells. (c) Representative western blotting analysis of uPAR, Ets-1, MMP-9, RhoC and TIMP-2 protein expression regulated by PRDX6 in MDA-MB-231 cells. (d) The relative expression of PRDX6 protein in the different MDA-MB-231 cells above was normalized to the signal intensity of β-actin. RT-PCR (e) and quantitative real-time PCR (f) analysis of uPAR, Ets-1, MMP-9, RhoC and TIMP-2 mRNA expression in different MDA-MB-435 cells. (g) Representative western blotting analysis of uPAR, Ets-1, MMP-9, RhoC and TIMP-2 protein expression in different MDA-MB-435 cells in vitro. (h) The relative expression of PRDX6 protein in the different MDA-MB-435 cells above was normalized to the signal intensity of β-actin. Click here for file

## References

[B1] Jemal A, Siegel R, Ward E, Murray T, Xu J, Thun MJ (2007). Cancer statistics, 2007. CA Cancer J Clin.

[B2] Li DQ, Wang L, Fei F, Hou YF, Luo JM, Zeng R, Wu J, Lu JS, Di GH, Ou ZL (2006). Identification of breast cancer metastasis-associated proteins in an isogenic tumor metastasis model using two-dimensional gel electrophoresis and liquid chromatography-ion trap-mass spectrometry. Proteomics.

[B3] Shichi H, Demar JC (1990). Non-selenium glutathione peroxidase without glutathione S-transferase activity from bovine ciliary body. Exp Eye Res.

[B4] Nagase T, Miyajima N, Tanaka A, Sazuka T, Seki N, Sato S, Tabata S, Ishikawa K, Kawarabayasi Y, Kotani H (1995). Prediction of the coding sequences of unidentified human genes. III. The coding sequences of 40 new genes (KIAA0081-KIAA0120) deduced by analysis of cDNA clones from human cell line KG-1 (supplement). DNA Res.

[B5] Kim TS, Sundaresh CS, Feinstein SI, Dodia C, Skach WR, Jain MK, Nagase T, Seki N, Ishikawa K, Nomura N (1997). Identification of a human cDNA clone for lysosomal type Ca2+-independent phospholipase A2 and properties of the expressed protein. J Biol Chem.

[B6] Kang SW, Baines IC, Rhee SG (1998). Characterization of a mammalian peroxiredoxin that contains one conserved cysteine. J Biol Chem.

[B7] Manevich Y, Fisher AB (2005). Peroxiredoxin 6, a 1-Cys peroxiredoxin, functions in antioxidant defense and lung phospholipid metabolism. Free Radic Biol Med.

[B8] Chang XZ, Wang ZM, Yu JM, Tian FG, Jin W, Zhang Y, Yu J, Li LF, Liu XF, Li ZW (2007). Isolation of a human gallbladder cancer cell clone with high invasive phenotype in vitro and metastatic potential in orthotopic model and inhibition of its invasiveness by heparanase antisense oligodeoxynucleotides. Clin Exp Metastasis.

[B9] Zhang Z, Futamura M, Vikis HG, Wang M, Li J, Wang Y, Guan KL, You M (2003). Positional cloning of the major quantitative trait locus underlying lung tumor susceptibility in mice. Proc Natl Acad Sci USA.

[B10] Albini A, Iwamoto Y, Kleinman HK, Martin GR, Aaronson SA, Kozlowski JM, McEwan RN (1987). A rapid in vitro assay for quantitating the invasive potential of tumor cells. Cancer Res.

[B11] Hou YF, Yuan ST, Li HC, Wu J, Lu JS, Liu G, Lu LJ, Shen ZZ, Ding J, Shao ZM (2004). ERbeta exerts multiple stimulative effects on human breast carcinoma cells. Oncogene.

[B12] Noh DY, Ahn SJ, Lee RA, Kim SW, Park IA, Chae HZ (2001). Overexpression of peroxiredoxin in human breast cancer. Anticancer Res.

[B13] Karihtala P, Mantyniemi A, Kang SW, Kinnula VL, Soini Y (2003). Peroxiredoxins in breast carcinoma. Clin Cancer Res.

[B14] Liu Y, Liu H, Han B, Zhang JT (2006). Identification of 14-3-3sigma as a contributor to drug resistance in human breast cancer cells using functional proteomic analysis. Cancer Res.

[B15] Wang T, Tamae D, LeBon T, Shively JE, Yen Y, Li JJ (2005). The role of peroxiredoxin II in radiation-resistant MCF-7 breast cancer cells. Cancer Res.

[B16] Iwao-Koizumi K, Matoba R, Ueno N, Kim SJ, Ando A, Miyoshi Y, Maeda E, Noguchi S, Kato K (2005). Prediction of docetaxel response in human breast cancer by gene expression profiling. J Clin Oncol.

[B17] Wang X, Phelan SA, Petros C, Taylor EF, Ledinski G, Jurgens G, Forsman-Semb K, Paigen B (2004). Peroxiredoxin 6 deficiency and atherosclerosis susceptibility in mice: significance of genetic background for assessing atherosclerosis. Atherosclerosis.

[B18] Krapfenbauer K, Yoo BC, Fountoulakis M, Mitrova E, Lubec G (2002). Expression patterns of antioxidant proteins in brains of patients with sporadic Creutzfeldt-Jacob disease. Electrophoresis.

[B19] Krapfenbauer K, Engidawork E, Cairns N, Fountoulakis M, Lubec G (2003). Aberrant expression of peroxiredoxin subtypes in neurodegenerative disorders. Brain Res.

[B20] Ouyang X, DeWeese TL, Nelson WG, Abate-Shen C (2005). Loss-of-function of Nkx3.1 promotes increased oxidative damage in prostate carcinogenesis. Cancer Res.

[B21] Neumann CA, Krause DS, Carman CV, Das S, Dubey DP, Abraham JL, Bronson RT, Fujiwara Y, Orkin SH, Van Etten RA (2003). Essential role for the peroxiredoxin Prdx1 in erythrocyte antioxidant defence and tumour suppression. Nature.

[B22] de Souza GA, Godoy LM, Teixeira VR, Otake AH, Sabino A, Rosa JC, Dinarte AR, Pinheiro DG, Silva WA, Eberlin MN (2006). Proteomic and SAGE profiling of murine melanoma progression indicates the reduction of proteins responsible for ROS degradation. Proteomics.

[B23] Quan C, Cha EJ, Lee HL, Han KH, Lee KM, Kim WJ (2006). Enhanced expression of peroxiredoxin I and VI correlates with development, recurrence and progression of human bladder cancer. J Urol.

[B24] Kinnula VL, Lehtonen S, Sormunen R, Kaarteenaho-Wiik R, Kang SW, Rhee SG, Soini Y (2002). Overexpression of peroxiredoxins I, II, III, V, and VI in malignant mesothelioma. J Pathol.

[B25] Soini Y, Kallio JP, Hirvikoski P, Helin H, Kellokumpu-Lehtinen P, Kang SW, Tammela TL, Peltoniemi M, Martikainen PM, Kinnula VL (2006). Oxidative/nitrosative stress and peroxiredoxin 2 are associated with grade and prognosis of human renal carcinoma. Apmis.

[B26] Wiegand S, Dunne AA, Muller HH, Mandic R, Barth P, Davis RK, Werner JA (2005). Metaanalysis of the significance of matrix metalloproteinases for lymph node disease in patients with head and neck squamous cell carcinoma. Cancer.

[B27] Wang Y (2001). The role and regulation of urokinase-type plasminogen activator receptor gene expression in cancer invasion and metastasis. Med Res Rev.

[B28] Pillay V, Dass CR, Choong PF (2007). The urokinase plasminogen activator receptor as a gene therapy target for cancer. Trends Biotechnol.

[B29] Kim HJ, Park CI, Park BW, Lee HD, Jung WH (2006). Expression of MT-1 MMP, MMP2, MMP9 and TIMP2 mRNAs in ductal carcinoma in situ and invasive ductal carcinoma of the breast. Yonsei Med J.

[B30] Lincoln DW, Bove K (2005). The transcription factor Ets-1 in breast cancer. Front Biosci.

[B31] Kleer CG, Griffith KA, Sabel MS, Gallagher G, van Golen KL, Wu ZF, Merajver SD (2005). RhoC-GTPase is a novel tissue biomarker associated with biologically aggressive carcinomas of the breast. Breast Cancer Res Treat.

