# Correction to: Identification of the functional role of peroxiredoxin 6 in the progression of breast cancer

**DOI:** 10.1186/s13058-018-0984-0

**Published:** 2018-07-02

**Authors:** Xin-Zhong Chang, Da-Qiang Li, Yi-Feng Hou, Jiong Wu, Jin-Song Lu, Gen-Hong Di, Wei Jin, Zhou-Luo Ou, Zhen-Zhou Shen, Zhi-Ming Shao

**Affiliations:** 1 Breast Cancer Institute, Cancer Hospital, Department of Oncology, Shanghai Medical College, Institutes of Biomedical Science, Fudan University, Shanghai, 200032 People’s Republic of China; 2 Tianjin Medical University Cancer Institute and Hospital, Hexi district, Tianjin, 300060 China

## Correction

After the publication of this work [1] an error in Fig. 1c was brought to our attention: the Western blots for PRDX6 and β-actin were similar to those shown in lanes 5-6 of Fig. 4g. To verify these findings, we have repeated this experiment and the results are shown in a new Fig. 1c below. The repeated experimental results are consistent with the previously reported findings in the original study [1] and the functional role for PRDX6 in malignant progression of human cancer including breast cancer has been widely documented and recognized in numerous other studies [2]. We apologize for the error. However, this correction does not affect the conclusions of the article.

**Fig. 1:**
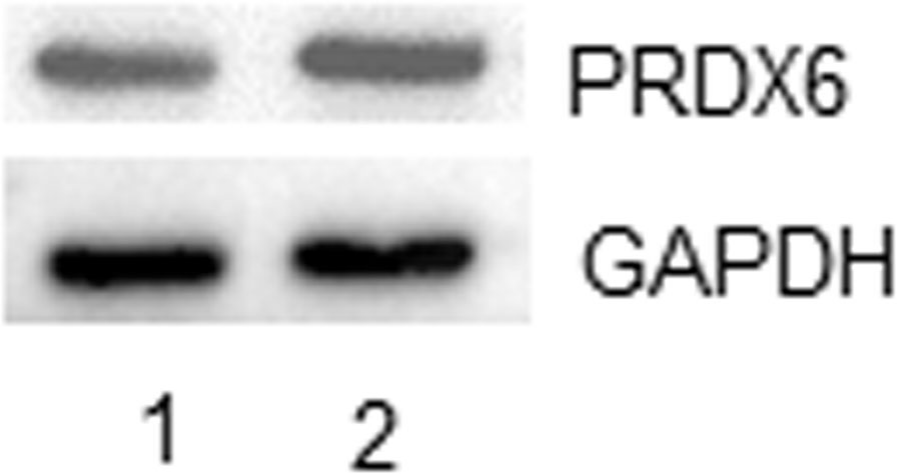
**c** Western blot analysis of PRDX6 and GAPDH expression in MDA-MB-231HM (lane 2) and parental MDA-MB-231 cell line (lane 1)
