# Supplementary material for: Identification of the functional role of peroxiredoxin 6 in the progression of breast cancer
Source: Breast Cancer Res. 2007 Nov 2;9(6):R76. doi: 10.1186/bcr1789 (PMC2246172; doi:10.1186/bcr1789)
Supplement: Additional file 1 — Table listing primers for RT-PCR and their annealing temperatures. [file bcr1789-S1.doc]

Supplementary Table 1.

Primers for RT-PCR and their annealing temperatures

| Gene |  | Primer sequence | Product size (bp) | Annealing (^0^C) |
| --- | --- | --- | --- | --- |
| cyclin A | Up | 5'-TCCATGTCAGTGCTGAGAGGA-3' |  |  |
|  | Down | 5'-GAAGGTCCATGAGACAAGGC-3' | 451 | 58 |
| cyclinD1 | Up | 5'-CTGGCCATGAACTACCTGGA-3' |  |  |
|  | Down | 5'-GTCACACTTGATCACTCTGG-3' | 482 | 51 |
| cyclin E | Up | , 5'-AATAGAGAGGAAGTCTGG-3' |  |  |
|  | Down | 5'-AGATATGCAACCTGCATG-3' | 442 | 60 |
| p21 | Up | 5'-ACTGTGATGCGCTAATGGC-3' |  |  |
|  | Down | 5'-ATGGTCTTCCTCTGCTGTCC-3' | 232 | 60 |
| MMP1 | Up | 5'-TTCATTTCTGTTTTCTGGCC-3' |  |  |
|  | Down | 5'-ATTTTTCCTGCAGTTGAACC-3' | 462 | 52 |
| MMP2 | Up | 5'-CAGGCTCTTCTCCTTTCACAAC-3' |  |  |
|  | Down | 5'-AAGCCACGGCTTGGTTTTCCTC-3' | 398 | 55 |
| MMP7 | Up | 5'-GTTTAG,AAGCCAAACTCAAGG--3' |  |  |
|  | Down | 5'-CTTTGACACTAATCGATCCAC-3' | 232 | 55 |
| MMP9 | Up | 5'-TGGGCTACGTGACCTATGACAT-3' |  |  |
|  | Down | 5'-GCCCAGCCCACCTCCACTCCTC-3' | 150 | 60 |
| Ets-1 | Up | 5'-GGGTGACGACTTCTTGTTTG -3' |  |  |
|  | Down | 5'-GTTAATGGAGTCAACCCAGC-3' | 274 | 57 |
| uPA | Up | 5'-GTGGCCAAAAGACTCTGAGG-3' |  |  |
|  | Down | 5'-GGCAGGCAGATGGTCTGTAT-3' | 400 | 58 |
| uPAR | Up | 5'-AGCTATCGGACTGGCTTGAA-3' |  |  |
|  | Down | 5'-TGTTGCAGCATTTCAGGAAG-3' | 352 | 55 |
| CathepsinD | Up | 5'-GACACAGGCACTTCCCTCAT-3' |  |  |
|  | Down | 5'-GTAGTAGCGGCCGATGAAGA-3' | 300 | 55 |
| Maspin | Up | 5'-CCCTATGCAAAGGAATTGGA-3' |  |  |
|  | Down | 5'-CAAGCCTGTGGACTCATCCT-3' | 399 | 57 |
| cystatin C | Up | 5'-CCAGCAACGACATGTACCAC-3' |  |  |
|  | Down | 5'-AAGGCACAGCGTAGATCTGG-3' | 207 | 57 |
| VEGF | Up | 5'-CTACCTCCACCATGCCAAGT-3' |  |  |
|  | Down | 5'-TCTCTCCTATGTGCTGGCCT-3' | 311 | 60 |
| bFGF | Up | 5'-AGAGCGACCCTCACATCAAG-3' |  |  |
|  | Down | 5'-ACTGCCCAGTTCGTTTCAGT-3' | 234 | 60 |
| Rho C | Up | 5'-ATGGCTGCAATCCGAAAGAAAG-3' |  |  |
|  | Down | 5'-TCAGAGAATGGGACAGCCCCT-3' | 582 | 55 |
| ps2 | Up | 5'-ggagaacaaggtgatctgcg-3 |  |  |
|  | Down | 5'-cacactcctcttctggaggg-3 | 363bp | 60 |
| TIMP-1 | Up | 5'-TTCGTGGGGACACCAGAAGTCAAC-3' |  |  |
|  | Down | 5'-TGGACACTGTGCAGGCTTCAGTTC-3' | 528 | 60 |
| TIMP-2 | Up | 5'-CTCGGCAGTGTGTGGGGTC-3' |  |  |
|  | Down | 5'-CGAGAAACTCCTGCTTGGGG-3' | 365 | 60 |
| TGFα | Up | 5'-GTGGTCTGAAGAGCCCAGAG-3' |  |  |
|  | Down | 5'-AACTGCTGCACACACCTCAC-3' | 302 | 57 |
| TPM4 | Up | 5′-ACGGTTGCAAAACTGGAAAA-3′ |  |  |
|  | Down | 5′-TTGGCTCTGGATGGAAAATC-3′ | 340 | 58 |
| E-cadherin | Up | 5′-AGCCATGGGCCCTTGGA-3′ |  |  |
|  | Down | 5′-CCAGAGGCTCTGTCACCTTC-3′ | 653 | 60 |
| C-jun | Up | 5'-GCATGAGGAACCGCATCGCTGCCTCCAAGT-3` |  |  |
|  | Down | 5'-GCGACCAAGTCCTTCCCACTCGTGCACACT-3` | 409 | 57 |
| C-fos | Up | 5`-AAGGAGAATCCGAAGGGAAAGGAATAAGATGGCT-3 |  |  |
|  | Down | 5'-AGACGAAGGAAGACGTGTAAGCAGTGCAGCT -3' | 612 | 55 |
| IGF-1 | Up | 5'-CTAGGCACTCTGCTTGC -3' |  |  |
|  | Down | 5'-CTTGGGCATGTCAGTGTGGC-3' | 296 | 55 |
| IGF-1R | Up | 5'-AACCACGAGGCTGAGAAGCT-3' |  |  |
|  | Down | 5'-CAGCATAATCACCAACCCTC-3' | 447 | 58 |
| IGF-2 | Up | 5'-ATGGGAATGCCAATGGGGAAG-3' |  |  |
|  | Down | 5'-CTTGCCCACGGGGTATCTGGG-3' | 236 | 55 |
| trophinin | Up | AGGGAAGAGTTAGGCGATGAT |  |  |
|  | Down | TTGGGCTCTGGCCTCAATT | 69 | 57 |
| GADPH | Up | 5'-GGGAGCCAAAAGGGTCATCATCTC-3' |  |  |
|  | Down | 5'-CCATGCCAGTGAGCTTCCCGTTC-3' | 353 | 60 |
