# Supplementary figures and images for: Identification of the functional role of peroxiredoxin 6 in the progression of breast cancer
Source: Breast Cancer Res. 2007 Nov 2;9(6):R76. doi: 10.1186/bcr1789 (PMC2246172; doi:10.1186/bcr1789)

# Supplementary Figure 1

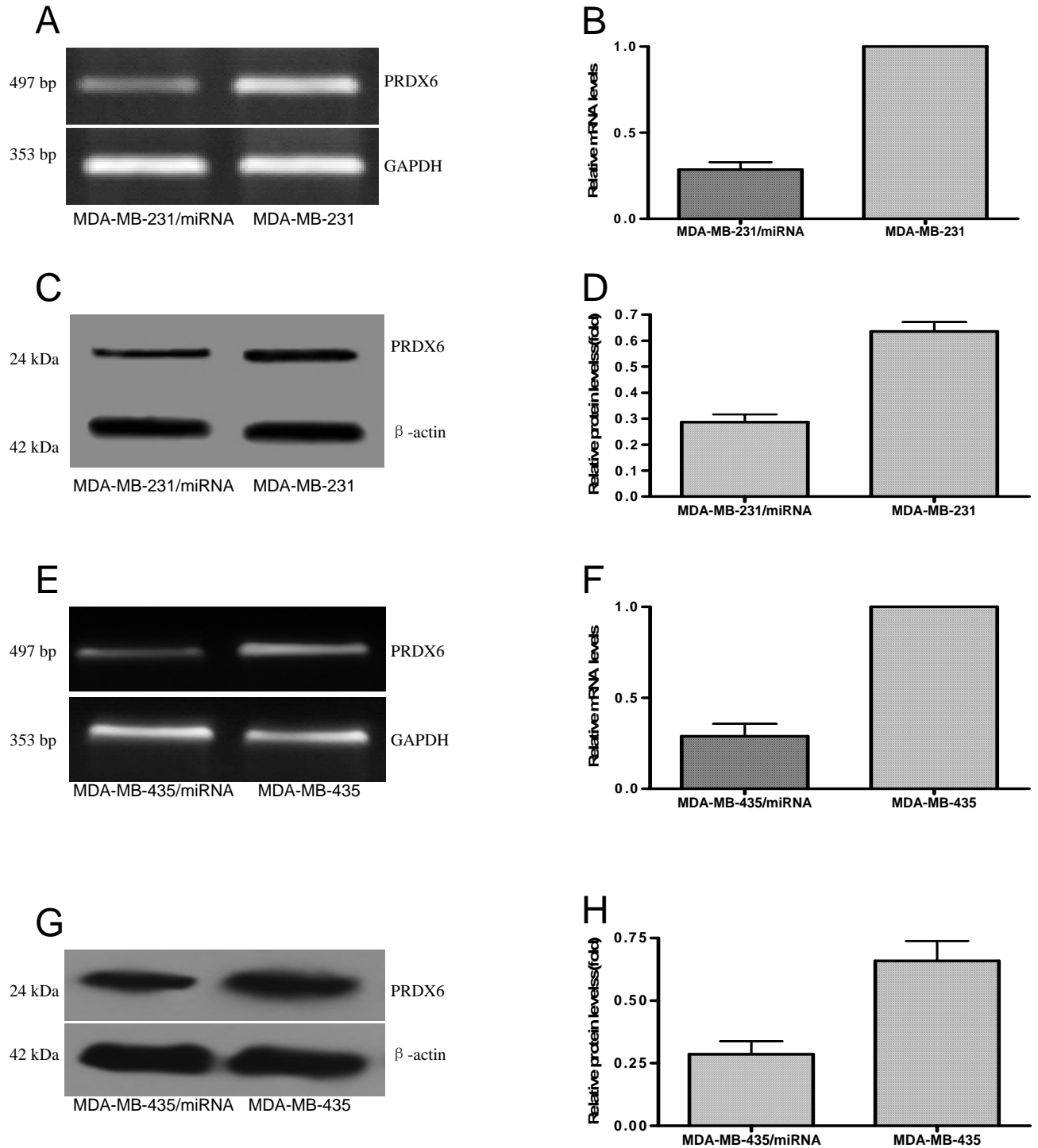

Supplement: Additional file 2 — Figure showing that peroxiredoxin (PRDX) 6 was inhibited in breast cancer cells at 3 months after initial transfection. RT-PCR (a) and real-time PCR (b) showed that pCMV-PRDX6 miRNA-672 decreased PRDX6 mRNA in MDA-MB-231 cells at 3 months after initial transfection. (C) Western blot analysis of PRDX6 in different MDA-MB-231 cells at 3 months after initial transfection. (d) The relative protein expressions of PRDX6 showed that pCMV-PRDX6 miRNA-672 decreased the PRDX6 protein in MDA-MB-231 cells at 3 months after initial transfection. RT-PCR (e) and real-time PCR (f) illustrated that pCMV-PRDX6 miRNA-672 decreased PRDX6 mRNA in MDA-MB-435 cells at 3 months after initial transfection. (g) Western blot analysis of PRDX6 in different MDA-MB-435 cells. (h) The relative protein expressions of PRDX6 showed that pCMV-PRDX6 miRNA-672 decreased PRDX6 protein in MDA-MB-435 cells. [file bcr1789-S2.pdf]

# Supplementary Figure 2

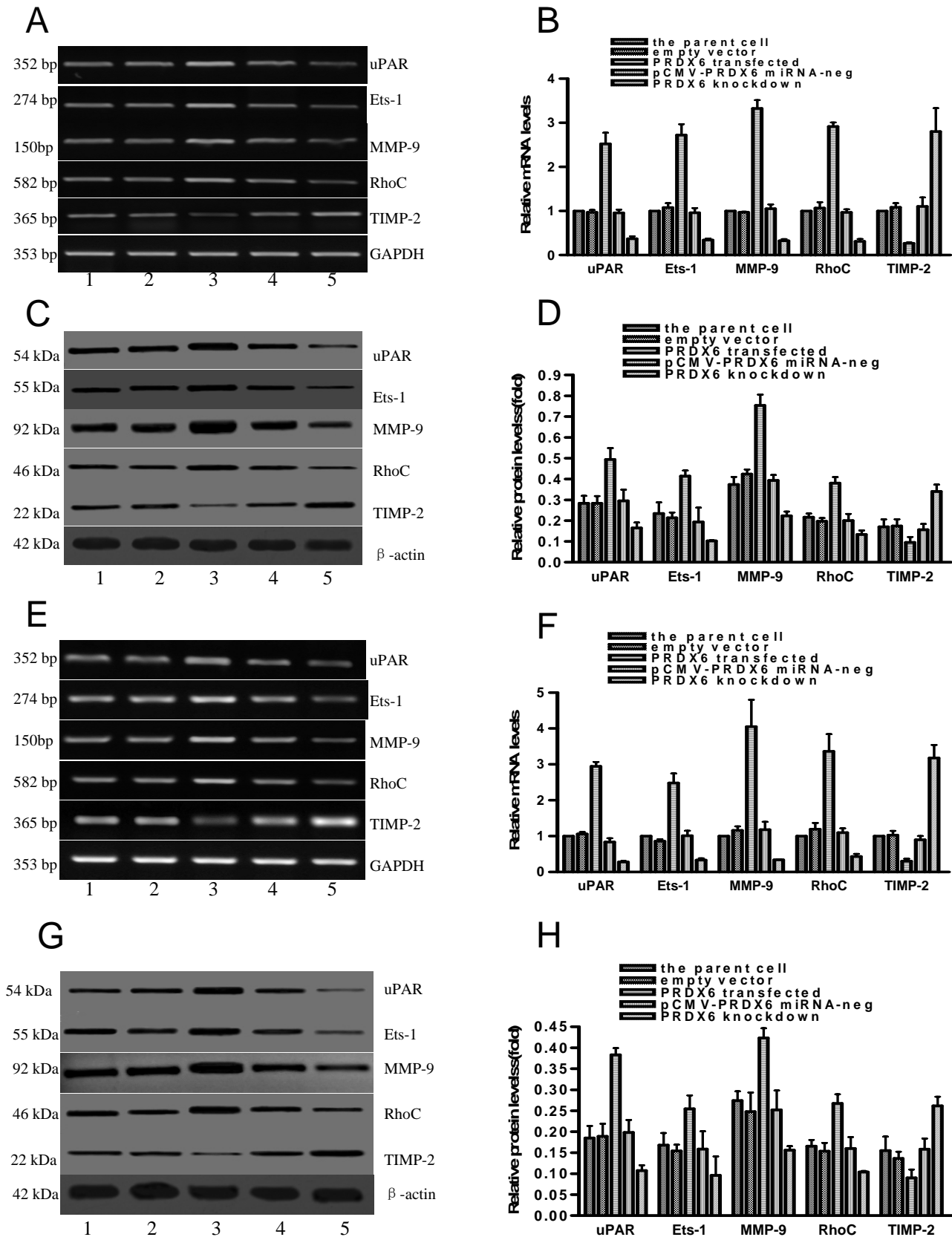

Supplement: Additional file 3 — Expression of peroxiredoxin (PRDX) 6 in breast cancer cells regulated urokinase-type plasminogen activator (uPA) receptor (uPAR), Ets-1, matrix metalloproteinase (MMP)-9, RhoC and tissue inhibitor of MMP (TIMP)-2 expression in vitro. Lanes 1 to 5: parental, empty vector, PRDX6-transfected, pCMV-PRDX6 miRNA-neg and PRDX6 knockdown cells. RT-PCR (a) and quantitative real-time PCR (b) analysis of uPAR, Ets-1, MMP-9, RhoC and TIMP-2 mRNA expression regulated by PRDX6 in MDA-MB-231 cells. (c) Representative western blotting analysis of uPAR, Ets-1, MMP-9, RhoC and TIMP-2 protein expression regulated by PRDX6 in MDA-MB-231 cells. (d) The relative expression of PRDX6 protein in the different MDA-MB-231 cells above was normalized to the signal intensity of β-actin. RT-PCR (e) and quantitative real-time PCR (f) analysis of uPAR, Ets-1, MMP-9, RhoC and TIMP-2 mRNA expression in different MDA-MB-435 cells. (g) Representative western blotting analysis of uPAR, Ets-1, MMP-9, RhoC and TIMP-2 protein expression in different MDA-MB-435 cells in vitro. (h) The relative expression of PRDX6 protein in the different MDA-MB-435 cells above was normalized to the signal intensity of β-actin. [file bcr1789-S3.pdf]
